# Utility of promoter hypermethylation in malignant risk stratification of intraductal papillary mucinous neoplasms

**DOI:** 10.1186/s13148-023-01429-5

**Published:** 2023-02-20

**Authors:** Ankit Chhoda, Anup Sharma, Bethsebie Sailo, Haoyu Tang, Nensi Ruzgar, Wan Ying Tan, Lee Ying, Rishabh Khatri, Anand Narayanan, Shrikant Mane, Bony De Kumar, Laura D. Wood, Christine Iacobuzio-Donahue, Christopher L. Wolfgang, John W. Kunstman, Ronald R. Salem, James J. Farrell, Nita Ahuja

**Affiliations:** 1 Division of Gastroenterology, Beth Israel Deaconess Medical Center, Harvard Medical School, Boston, USA; 2 Department of Surgery, Yale School of Medicine, New Haven, USA; 3 Yale Systems Biology Institute, Yale University, New Haven, USA; 4 Department of Internal Medicine, Temple University Hospital, Philadelphia, USA; 5 Yale Center for Genome Analysis, New Haven, USA; 6 Department of Pathology, Johns Hopkins University, Baltimore, USA; 7 Department of Pathology, Memorial Sloan Kettering, New York City, USA; 8 Department of Surgery, NYU Langone Health, New York City, USA; 9 Section of Digestive Diseases, Department of Internal Medicine, Yale School of Medicine, New Haven, USA; 10 Department of Pathology, Yale School of Medicine, New Haven, USA; 11 Department of Biological and Biomedical Sciences, Yale School of Medicine, New Haven, USA

**Keywords:** Pancreatic cyst, Pancreatic cancer, Methylation-specific biomarker

## Abstract

**Background:**

Intraductal papillary mucinous neoplasms (IPMNs), a type of cystic pancreatic cancer (PC) precursors, are increasingly identified on cross-sectional imaging and present a significant diagnostic challenge. While surgical resection of IPMN-related advanced neoplasia, i.e., IPMN-related high-grade dysplasia or PC, is an essential early PC detection strategy, resection is not recommended for IPMN-low-grade dysplasia (LGD) due to minimal risk of carcinogenesis, and significant procedural risks. Based on their promising results in prior validation studies targeting early detection of classical PC, DNA hypermethylation-based markers may serve as a biomarker for malignant risk stratification of IPMNs. This study investigates our DNA methylation-based PC biomarker panel (*ADAMTS1, BNC1*, and *CACNA1G* genes) in differentiating IPMN-advanced neoplasia from IPMN-LGDs.

**Methods:**

Our previously described genome-wide pharmaco-epigenetic method identified multiple genes as potential targets for PC detection. The combination was further optimized and validated for early detection of classical PC in previous case–control studies. These promising genes were evaluated among micro-dissected IPMN tissue (IPMN-LGD: 35, IPMN-advanced neoplasia: 35) through Methylation-Specific PCR. The discriminant capacity of individual and combination of genes were delineated through Receiver Operating Characteristics curve analysis.

**Results:**

As compared to IPMN-LGDs, IPMN-advanced neoplasia had higher hypermethylation frequency of candidate genes: *ADAMTS1* (60% vs. 14%), *BNC1* (66% vs. 3%), and *CACGNA1G* (25% vs. 0%). We observed Area Under Curve (AUC) values of 0.73 for *ADAMTS1*, 0.81 for *BNC1*, and 0.63 for *CACNA1G* genes. The combination of the *BNC1/ CACNA1G* genes resulted in an AUC of 0.84, sensitivity of 71%, and specificity of 97%. Combining the methylation status of the *BNC1/CACNA1G* genes, blood-based CA19-9, and IPMN lesion size enhanced the AUC to 0.92.

**Conclusion:**

DNA-methylation based biomarkers have shown a high diagnostic specificity and moderate sensitivity for differentiating IPMN-advanced neoplasia from LGDs. Addition of specific methylation targets can improve the accuracy of the methylation biomarker panel and enable the development of noninvasive IPMN stratification biomarkers.

**Supplementary Information:**

The online version contains supplementary material available at 10.1186/s13148-023-01429-5.

## Background

Intraductal papillary mucinous neoplasms (IPMNs), a type of cystic pancreatic cancer (PC) precursors, are increasingly identified on cross-sectional imaging and present a significant diagnostic challenge [1]. IPMNs necessitate accurate differentiation from non-precancerous lesions (such as pseudocysts, and serous cystadenoma) as well as malignant risk stratification. Histologically, resection of IPMN-associated advanced neoplasia including high-grade dysplasia (HGD) or pancreatic cancer (PC) and attainment of negative margins, has been demonstrated to improve patient prognosis and, therefore, is a critical early PC detection strategy—[2]. The resection of IPMN-low-grade dysplasia (LGD) is not recommended due to significant procedural risks and minimal risk of developing invasive PC. Stratification inaccuracies may culminate in false-negative results risking interval IPMN progression, while false positives may lead to unnecessary surgical resections. IPMN stratification into LGD and advanced neoplasia is a critical domain which warrants dedicated research and has been the focus of this study.

Previously, clinical-morphologic features, such as cyst size, CA19-9 levels, etc., have been utilized to attempt classification of IPMNs into those with worrisome features or high-risk stigmata and the remaining low-risk IPMNs [3–5]. Currently accepted Fukuoka consensus guidelines have low sensitivity and specificity (sensitivity 56–81%; specificity: 69–73%), and evidence accrued over the years has demonstrated that IPMN stratification-based solely on cyst morphology is less than ideal [6]. Augmentation of the accuracy of morphologic stratification with additional biomarkers has been advocated for early detection of prognostically significant IPMN-related advanced neoplasia from LGD.

Hypermethylation of promoter CpG islands occurs early in pancreatic carcinogenesis and may cause silencing of tumor suppressor genes [7]. Our previous work demonstrated promising results in the detection of classical PC by utilizing hypermethylation of candidate genes identified using a genome-wide pharmaco-epigenetic approach with high diagnostic sensitivity (81–97.4%) and specificity (85–91.6%) [8, 9]. Our ongoing work has investigated the utility of DNA methylation-based biomarker strategy for stratification of precancerous lesions. The current study investigates the diagnostic utility of aberrant DNA methylation of A Disintegrin-like Metalloproteinase with Thrombospondin type 1 motif 1 (*ADAMTS1*), Basonuclein 1 (*BNC1*), and T-type calcium channel (*CACNA1G*) genes in differentiating histologically defined IPMN-related advanced neoplasia from IPMN-LGDs.

## Results

### Patient demographics

The study analyzed a random selection of 70 tissue samples from IPMN patients who underwent surgical resection, matched 1:1 IPMN-LGD versus IPMN-advanced neoplasia. Patients met the criteria for surgical resection based on currently available clinical guidelines [3–5]. The overall study population had a median age of 71.5 years, predominately male 51.3%, and White 92.9% (Table 1). The median size of the lesion resection was 3 cm, and 18.6% of lesions had elevated blood carbohydrate antigen 19-9 (CA19-9, ≥ 37 U/mL). A history of current and past smoking was present in 15.7% and 27.1% of patients, respectively. Most patients (77.6%) underwent a pancreaticoduodenectomy. The IPMN-advanced neoplasia and LGD groups were comparable in terms of sex distribution (female: 51.4 vs. 45.7%), smoking history (20% vs. 11.4%), and age distribution (72.5 years vs. 70.0 years) (Table 1). However, IPMN-related advanced neoplasia had a larger size (3.3 cm vs. 2.5 cm; *P* < 0.05) and CA19-9 positivity (31.4% vs. 5.7%; *P* < 0.01) when compared to IPMN-LGD.

**Table 1:** Baseline study population demographics

Demographics	Total	IPMN advanced neoplasia	IPMN-LGD	*p*-value
	*n* = 70	*n* = 35	*n* = 35	
*Sex distribution: n (%)*				
Female	34 (48.7%)	18 (51.4%)	16 (45.7%)	0.6
*Racial distribution: n (%)*				
White	65 (92.9%)	31 (88.6%)	34 (97.1%)	
Black	1 (1.4%)	0	1 (2.9%)	–
Others	1 (1.4%)	1 (2.9%)	0	
Unknown	3 (4.3%)	3 (8.6)	0	
*Smoking history: n (%)*				
Current	11 (15.7%)	7 (20%)	4 (11.4%)	0.5
Past	19 (27.1%)	10 (28.8%)	9 (25.7%)	
Never	40 (57.2%)	18 (51.4%)	22 (62.9%)	
*Age at diagnosis*				
Median (IQR)	71.5 (65.2–75)	72.5 (67.0–76.5)	70.0 (62.0–74.0)	0.8
*Surgical resection: n (%)*				
Distal pancreatectomy	11 (15.4%)	5 (14.3%)	6 (17.1%)	
Pancreaticoduodenectomy	54 (77.6%)	26 (74.3%)	28 (80.0%)	
Total pancreatectomy	2 (2.8%)	2 (5.7%)	0	–
Enucleation	1 (1.4%)	0	1 (2.9%)	
Unknown	2 (2.8%)	2 (5.7%)	0	
CA19-9 positive (≥ 37 U/mL): *n* (%)	13 (18.6%)	11 (31.4%)	2 (5.7%)	< 0.01
IPMN lesion size (median, IQR)^#^	3.0 (1.8–3.7)	3.3 (2.5–4.3)	2.5 (1.5–3.0)	< 0.01

*IQR* Interquartile Range, *CA19-9* Carbohydrate antigen 19-9

^#^Lesion size data unavailable for 6 patients, –: insufficient values

### Hypermethylation of *ADAMTS1, BNC1*, and *CACNA1G* genes among IPMNs

**Fig. 1:**
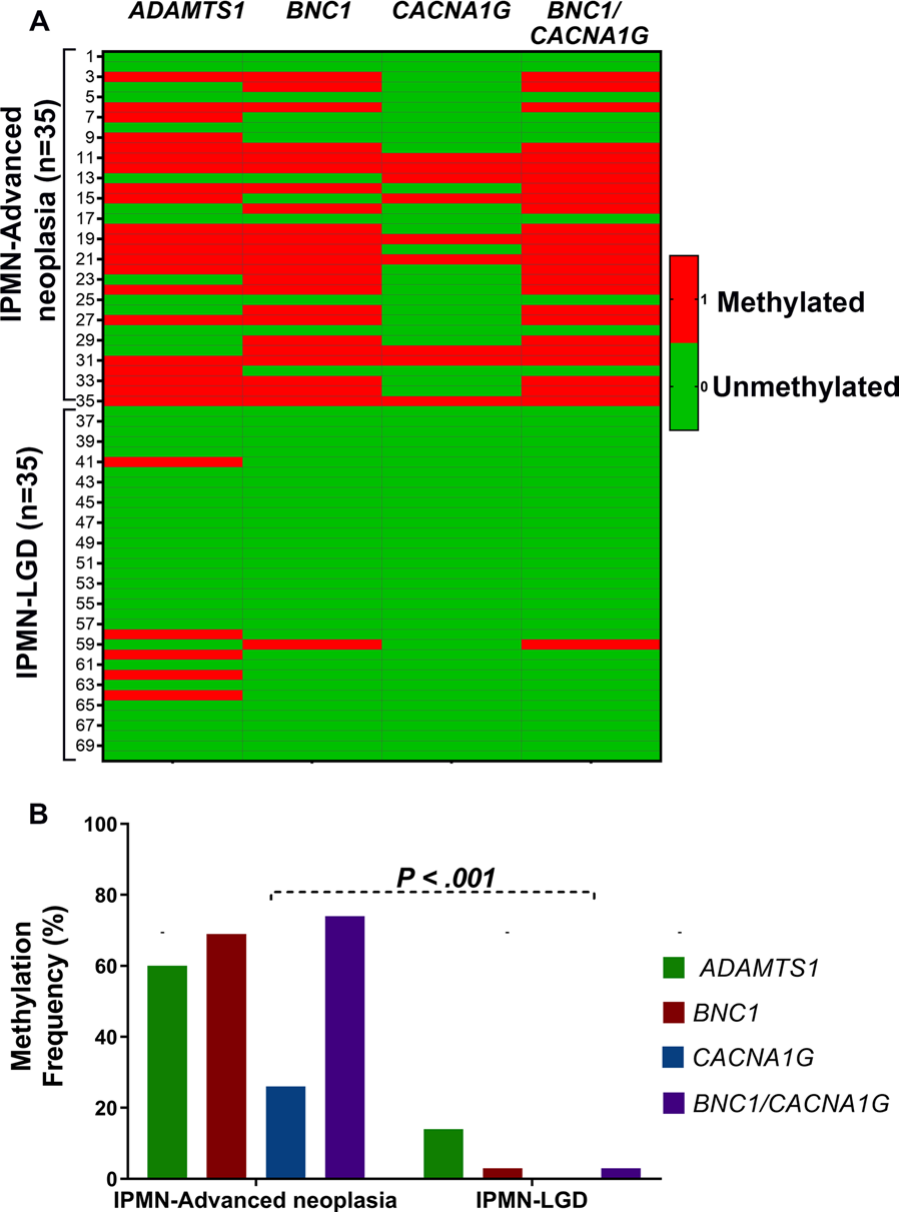
Methylation frequency of hyper methylated genes in IPMN and/or advanced neoplasia: **A** heat map of individual genes among IPMN-LGD versus IPMN-advanced neoplasia (red—methylated, green—unmethylated) in respective patient. **B** Methylation frequency among various genes in IPMN tissue samples

In the methods section, we describe conventional and quantitative methylation-specific polymerase chain reaction (MS-PCR), which enabled the calculation of CpG island methylation in the promoter region of *ADAMTS1, BNC1,* and *CACNA1G* genes. Each gene was analyzed for methylation frequency in IPMN-advanced neoplasia and IPMN-LGD tissue samples (Fig. 1A). IPMN-related advanced neoplasia had significantly higher methylation of *ADAMTS1* gene as compared to IPMN-LGD (60% vs. 14%, *P* < 0.001). *BNC1* gene had significantly higher methylation frequency among IPMN-related advanced neoplasia compared to IPMN-LGD (66% vs. 3%, *P* < 0.001). *CACNA1G* genes were methylated in 0% of IPMN-LGDs and 25% of IPMN-related advanced neoplasia. Overall, DNA methylation frequency among IPMN-advanced neoplasia was significantly higher than among IPMN-LGDs (80% vs. 27%, *P* < 0.001) (Fig. 1B).

### Diagnostic accuracy of DNA methylation-based biomarker among IPMN tissue

**Fig. 2:**
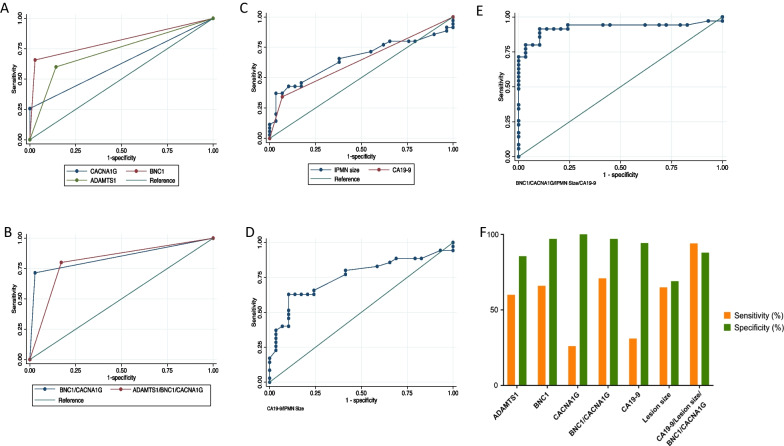
Receiver operator curve (ROC) analysis investigating the predictive capacity of: **A** Promoter hypermethylation of *ADAMTS1, BNC1, *and *CACNA1G* genes. **B** Combination of *ADAMTS1/BNC1/CACNA1G *and* BNC1/CACNA1G* genes. (C) IPMN lesion size and CA19-9 levels. **D** Combination of IPMN lesion size (continuous variable) and CA19-9. **E** Combination of promoter hypermethylation of *BNC1/CACNA1G* genes, IPMN size, and CA19-9 in the prediction of advanced neoplasia. **F** Diagnostic sensitivity and specificity of methylation status of individual genes, CA19-9, cyst features, and their combination

We observed that the promoter hypermethylation of *CACNA1G, ADAMTS1*, and *BNC1* genes had overall diagnostic accuracy, i.e., AUCs of 0.63 (0.50–0.74), 0.73 (0.61–0.83), and 0.81 (0.070–0.90), respectively, in distinguishing IPMN-related advanced neoplasia from IPMN-LGD (Fig. 2A; Table 2). As previously demonstrated, the aggregation of multiple genes into biomarker panels improves predictive power [8, 9]. A combination of *BNC1/CACNA1G* genes achieved an AUC of 0.84 (0.74–0.92. *P* < 0.001) (Fig. 2B, Table 2). IPMN lesion size as a continuous parameter, had an AUC of 0.66 (0.53–0.77) (Fig. 2C, Table 2). The conventional blood-based biomarker CA19-9 (≥ 37 U/mL) which is also a worrisome feature per Fukuoka guidelines had an AUC of 0.61 (0.50–0.72) (Fig. 2C, Table 2). By using CA19-9 and IPMN size, an AUC of 0.75 was achieved (Fig. 2D, Table 2). A combination of cyst features lesion size, CA19-9, and methylation status of *BNC1/CACNA1G* genes demonstrated an AUC of 0.92 (0.86–0.98) (Fig. 2E, Table 2).

**Table 2:** Diagnostic accuracy of various methylation biomarker panel

Biomarker	Area under the curve	*P*-value	Sensitivity (%)	Specificity (%)
*ADAMTS1*	0.73 (0.61–0.83)	0.001	60	86
*BNC1*	0.81 (0.70–0.90)	< 0.001	66	97
*CACNA1G*	0.63 (0.50–0.74)	0.06	26	100
*ADAMTS1/BNC1*	0.80 (0.69–0.89)	< 0.001	77	83
*ADAMTS1/BNC1/CACNA1G*	0.81 (0.70–0.90)	< 0.001	83	80
*BNC1/CACNA1G*	0.84 (0.74–0.92)	< 0.001	71	97
IPMN Lesion Size^#^	0.66 (0.53–0.77)	0.01	65	69
IPMN Lesion Size^#^ and CA19-9	0.75(0.65–0.86)	0.03	66	82
CA19-9	0.61 (0.50–0.72)	0.06	34	88
IPMN Lesion Size^#^, CA19-9, & *CACNA1G/BNC1*	0.92 (0.86–0.98)	< 0.001	94	88

*CA19-9* Carbohydrate antigen 19-9

^#^Continuous variable

The diagnostic sensitivity and specificity of *ADAMTS1* gene were 60% and 86%, *BNC1* gene were 66% and 97% and *CACNA1G* gene were 26% and 100%, respectively (Table 2, Fig. 2F). The combination of *BNC1/CACNA1G* genes achieved diagnostic sensitivity and specificity of 71% and 97%, respectively. Lesion size had a diagnostic sensitivity of 65% and specificity of 69% whereas CA19-9 had a sensitivity of 34% and specificity of 88%. The combination of lesion size, CA19-9, and methylation status of *BNC1/CACNA1G* genes, had a sensitivity of 94% and specificity of 88%.

## Discussion

Methylation-based biomarker strategies have emerged as important for early detection, including tests for stool DNA (*SEPT9*, Cologuard), liquid biopsies (EpiProColon), and urine-based strategies for renal tumors [10–12]. Our prior work demonstrated high accuracy in the detection of early-stage PCs (Stage 1 and II) in tissues as well as non-invasive liquid biopsies (sensitivity: 94.8% and specificity: 91.6%) [8, 9]. Hypermethylation of promoter CpG islands occurs early in pancreatic carcinogenesis and may cause silencing of tumor suppressor genes [7, 8, 13]. Promoter hypermethylation of *ADAMTS1* gene has a role in angiogenesis and cancer metastasis [14]. *BNC1* and *CACNA1G* genes regulate epithelial plasticity and cellular proliferation, respectively [15, 16].

At a tissue level, i.e., resected specimens, cyst fluid, and pancreatic juice, methylation-based markers have previously been investigated for IPMN stratification [17–24]. Some of the studies utilized methylation status of tumor suppressor genes (*WWOX*, *SMAD4*, and *CDO1* genes) [17, 23] or markers related to mucinous lineage (such as *GNAS, MUC* family) [19, 24]. Similar to our study, the investigators used a multigene approach to enhance diagnostic accuracy [20, 22]. However, advanced neoplasia was often compared to heterogeneous controls, including cysts with low/no malignant potential (pseudocysts, serous cystadenomas) or normal pancreatic tissue admixed with IPMN-LGD. The inclusion of normal tissue and non-precancerous lesions in control might have decreased the threshold of diagnostic performance and spurious elevation of AUCs [20, 22]. Utilization of only IPMN-LGD as controls caters to a more specific question of malignant stratification of IPMNs. In clinical practice, delineation of IPMNs from lesions such as serous cystadenoma and pancreatic pseudocysts can be achieved through cyst morphology and aspirate cytology [25, 26].

Cyst morphology-based stratification described in various consensus guidelines (such as Fukuoka, American Gastroenterology Association, and European guidelines) defined high-risk morphologic features such as high-risk stigmata, and worrisome features have less than perfect accuracy [5, 27]. Therefore, clinical decisions based solely on these guidelines risk unnecessary surgical resections as well as inadvertent interval malignant progression. Even in the current study, 35 IPMN-LGDs underwent surgical resection based on high-risk morphological features but were later observed to have benign histology. Combination of *BNC1*/*CACNA1G* genes had favorable diagnostic accuracy (AUC of 0.84, sensitivity of 71%, and specificity of 97%), which further improved when combined with IPMN lesion size (continuous variable) and CA19-9 (AUC: 0.92: sensitivity of 94%, and specificity of 88%). Thus, we endorse utilization of DNA methylation-based biomarkers in supplementing IPMN management decisions.

Our study investigates a scientifically important question of the malignant progression of IPMN, which is also clinically relevant. Each IPMN tissue in this study underwent laser microdissection at a high-volume multidisciplinary pancreatic cancer center and was thoroughly reviewed to ascertain mucinous lineage. Using methylation-specific PCR also ensures good analytical sensitivity and much higher specificity. However, the current study relied on histologically characterized tissue samples, making the test invasive. Furthermore, the study population was predominantly Caucasian and warrants further study in diverse population samples. IPMN cell lines, some of which have been described in the literature, were not readily available to us [28]. Thus, lack of cancer cell lines with mucinous lineage, which formed the basis of our biomarker discovery, adds to the limitations of this study.

## Conclusions

We demonstrated that highly specific methylation markers in *BNC1/CACNA1G* genes can be utilized to differentiate IPMN with advanced neoplasia from LGD lesions. Further multiplexing with specific methylation targets can further enhance the diagnostic sensitivity of the biomarker panel and pave the way for high-fidelity noninvasive biomarkers for IPMN stratification through blood-based, urine-based, or pancreatic cyst fluid assays.

## Methods

### Patients and specimens

This study received ethics approval from Institutional Review Board (IRB#2000022652) at Yale University. Samples were selected from a prospectively maintained database of IPMN patients. The IPMN patients underwent surgical resection based on clinical consensus guidelines in a high-volume multidisciplinary clinic [29]. The patients with Fukuoka-positive IPMN who underwent surgical resection were consented and their baseline demographic and histologic characteristics were recorded. The samples of IPMN with LGD versus IPMN with HGD/PC (*n* = 35 each) samples were matched 1:1 based on age, sex, smoking, and race (Table 1). The surgically removed IPMN tissue samples were graded by two expert pathologists (LW or CID) using a newly developed classification system for pancreatic neoplastic precursor lesions [30].

### Identification of methylation-specific novel biomarkers

We previously described the pharmaco-epigenomic identification of the *ADAMTS1/BNC1/CACN1G* genes and evaluated the diagnostic accuracy of this biomarker panel in detecting PC in both tissues and liquid samples. In brief, novel methylation-regulated genes were identified through the treatment of pancreatic cancer cell lines with DNA methyltransferase inhibitor: 5-aza-2′-deoxycytidine (DAC) and histone deacetylase inhibitor Trichostatin A (TSA). Gene expression was analyzed through transcriptome-wide Agilent 44 K Expression Array, and the genes which re-expressed with DAC but not TSA were then filtered to identify pancreatic cancer-specific genes and then confirmed in The Cancer Genome Atlas data (Additional file 1: Figure S1). We further show the methylation status of all three genes *ADMATS1, BNC1,* and *CACNA1G* in the TCGA dataset of pancreas cancer (Additional file 1: Figure S2) [8, 9]. The purpose of this study is to determine the use of a biomarker panel based on abnormal hypermethylation of the *ADAMTS1, BNC1*, and *CACNA1G* genes in the malignant classification of histologically defined IPMN tissue [8, 9, 31].

### Tissue DNA extraction and methylation analysis

Formalin-fixed paraffin-embedded tissue samples were evaluated using conventional MS-PCR, as described previously [32]. This project spanned over a decade and initially involved conventional MS-PCR for *CACNA1G*. To evaluate the methylation status of the *ADAMTS1* and *BNC1* genes, we used TaqMan probe-based PCR amplification (IDT Inc.) on bisulfite-converted DNA isolated from IPMN tissue using quantitative MS-PCR, as described in our previous publications [8, 9]. Both conventional MS-PCR (*CACNA1G*) and quantitative MS-PCR (*ADAMTS1/BNC1*) were used to determine promoter hypermethylation among tissue samples. For quantification, the comparative cycle threshold (*C*_t_) method was used, normalizing the *C*_t_ values for the candidate gene to the *C*_t_ values of unmethylated reaction relative to a methylated reaction sample. CpG methylated Jurkat genomic DNA (Thermofisher Scientific) was used as methylation positive control (Life Technologies) and β-actin as a housekeeping gene for normalization. Negative controls included non-template water samples. All studies followed the standards for the minimum information required for the publication of quantitative real-time PCR experiments.

### Statistical analysis

The continuous variables were described using medians and interquartile ranges, and categorical variables were described with frequencies. Mann–Whitney U and *χ*^2^ test were used to analyze the nonparametric continuous and categorical variables, respectively. The Receiver Operator Characteristic Curve (ROC) analysis (nonparametric model) was used to determine the diagnostic accuracy of each gene or their combination in predicting the presence of advanced neoplasia. The combination of lesional size, CA19-9, and results of DNA methylation markers were combined using logistic regression model. These models then underwent ROC analyses to determine discriminant capacity. STATA Version 17.0 was used for all statistical analysis (StataCorp LLC, College Station, Texas). The significant associations were then included in multivariate regression. A *P*-value of less than 0.05 was considered significant.

## Supplementary Information

**Additional file 1: Figure S1: **Study design and Identification of CACNA1G gene based on the pharmaco-epigenomic method. **Figure S2:** Methylation levels of (**A**) *ADAMTS1*, (**B**) *BNC1*, (**C**) *CACNA1G* genes in the Cancer Genome Atlas [Díez-Villanueva A, Mallona I, Peinado MA. Wanderer, an interactive viewer to explore DNA methylation and gene expression data in human cancer. Epigenetics Chromatin 2015;8:22. https://doi.org/10.1186/s13072-015-0014-8].

## Data Availability

All data generated or analyzed during this study are included in this published article.

## Abbreviations

AUC: Area under the curve
CA19-9: Carbohydrate antigen 19-9
HGD: High-grade dysplasia
IPMN: Intraductal papillary mucinous neoplasms
IQR: Interquartile range
LGD: Low-grade dysplasia
MS-PCR: Methylation-specific polymerase chain reaction
PC: Pancreatic cancer
