# Supplementary figures and images for: Utility of promoter hypermethylation in malignant risk stratification of intraductal papillary mucinous neoplasms
Source: Clin Epigenetics. 2023 Feb 20;15:28. doi: 10.1186/s13148-023-01429-5 (PMC9942382; doi:10.1186/s13148-023-01429-5)

## Slide 1
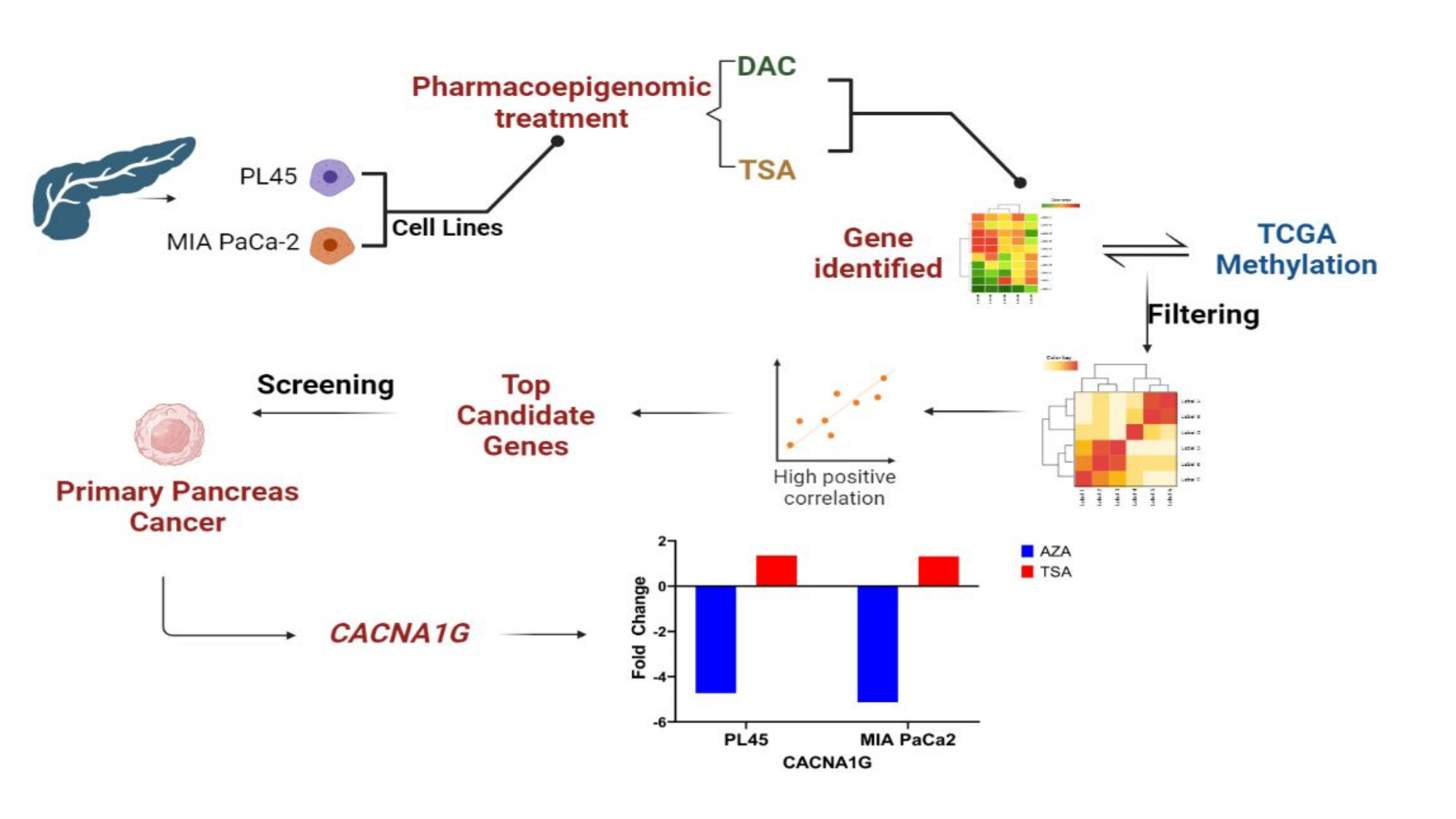

#

## Slide 2
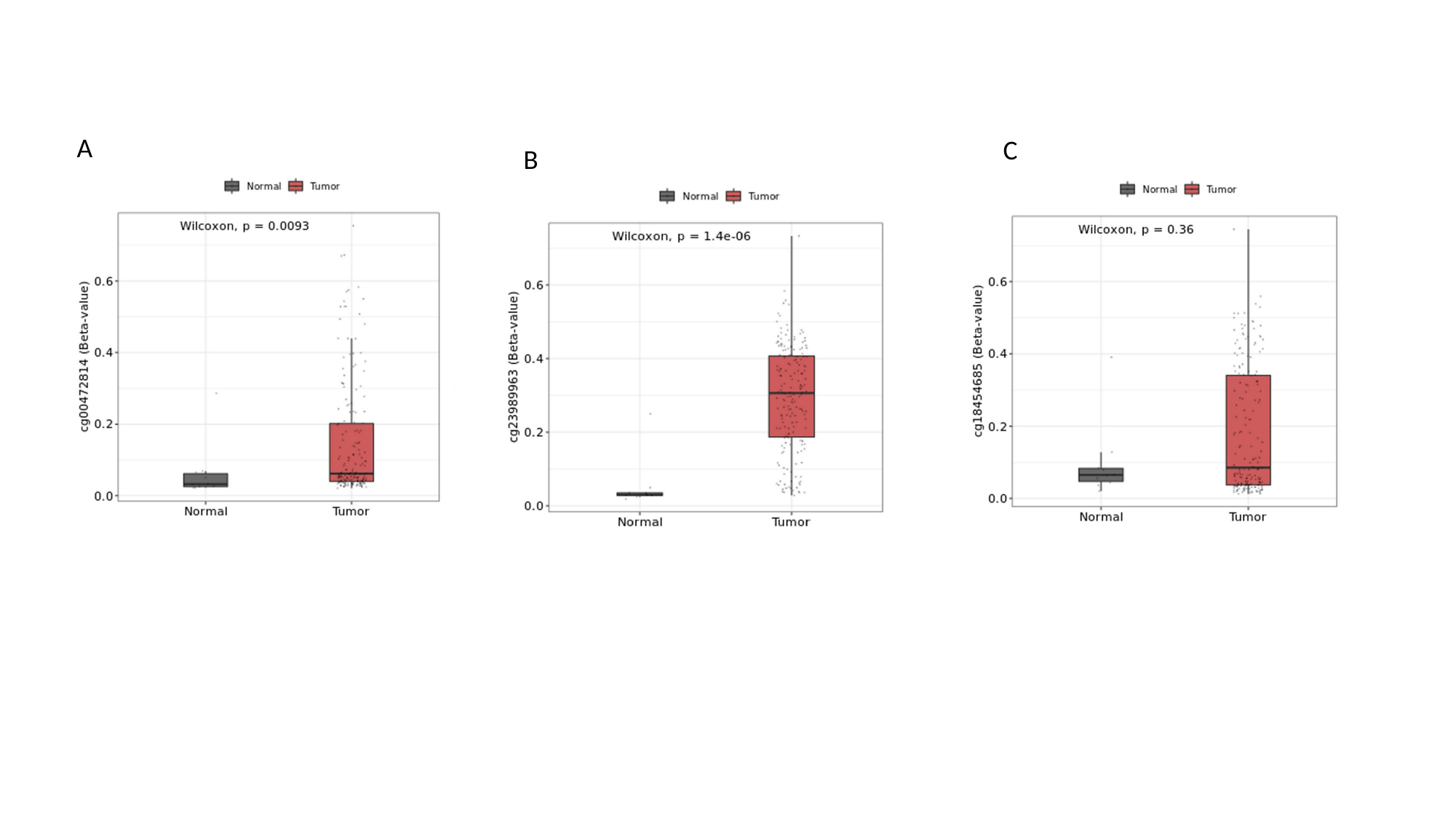

A
C
B

Supplement: Supplementary file 1 — Additional file 1: Figure S1: Study design and Identification of CACNA1G gene based on the pharmaco-epigenomic method. Figure S2: Methylation levels of (A) ADAMTS1, (B) BNC1, (C) CACNA1G genes in the Cancer Genome Atlas [Díez-Villanueva A, Mallona I, Peinado MA. Wanderer, an interactive viewer to explore DNA methylation and gene expression data in human cancer. Epigenetics Chromatin 2015;8:22. https://doi.org/10.1186/s13072-015-0014-8]. [file 13148_2023_1429_MOESM1_ESM.pptx]
